# Improving the Interdisciplinary Clinical Education of a Palliative Care Program through Quality Improvement Initiatives

**DOI:** 10.1089/pmr.2020.0092

**Published:** 2020-11-19

**Authors:** Meghan Thiel, Karen Harden, Lori-Jene Brazier, Adam Marks, Michael Smith

**Affiliations:** ^1^ Department of Internal Medicine, Division of Geriatric and Palliative Medicine, University of Michigan, Michigan Medicine, Ann Arbor, Michigan, USA.; ^2^ University of Michigan School of Nursing, Ann Arbor, Michigan, USA.; ^3^ University of Michigan School of Medicine, Ann Arbor, Michigan, USA.; ^4^ University of Michigan School of Pharmacy, Ann Arbor, Michigan, USA.

**Keywords:** clinical education, interdisciplinary education, interprofessional education, quality improvement

## Abstract

Palliative care relies on a team approach to manage the complex needs of patients and families living with serious illness. This article describes an interprofessional team's aim to develop an interactive online curriculum in palliative care, with an emphasis on interprofessional education (IPE). The aim of the program is to address the need for formalized interprofessional palliative care education. The interdisciplinary team identified the need for formalized education efforts within our clinical space. To address the need, the team designed an online curriculum based in the core competencies of palliative care and IPE. A new model was established, with the themes of learning about “people,” learning the “job,” and learning “respect.” The team followed the plan-do-study-act model to guide their process. The newly developed interprofessional online curriculum was utilized by palliative care trainees from various disciplines and levels of education. Pre- and post-tests to measure the knowledge, behavior, attitudes, and skills needed for teamwork and core palliative care competencies were completed. Forty-three medical and nursing students, undergraduate and graduate, completed the pretest, and 32 students completed the post-test. Results indicate that learners are growing in interprofessional skills and attitudes, but not in formalized knowledge of palliative care as a result of their clinical experience. Results suggest that more formalized knowledge may need to be provided to learners who come to this clinical experience, which could be delivered through the online curriculum. The knowledge survey should also be re-evaluated for clarity and content.

## Introduction

Medicine is no longer practiced in silos; interprofessional collaboration occurring across the spectrum of health care and emerging research supports this advancement.^1,2^ Recently, the National Consensus Project for Quality Palliative Care published guidelines for providing high-quality palliative care.^3^ These guidelines discuss and promote the interprofessional teamwork needed to take care of patients with serious illness. A section within these guidelines is dedicated to “interdisciplinary team education”; much of its focus is on ensuring the team members have the proper education and credentialing to work successfully as an interprofessional team. One statement, “Education, resources and support are available to enhance interdisciplinary team communication and collaboration,” provides an overview of what should be available to team members, but with little guidance.

The vast majority of efforts within interprofessional palliative care education have largely focused on student learners in coursework. The most well-known example is Interprofessional Curriculum for Oncology Palliative Care Education (iCOPE), which has been well described in this journal.^4^ This program was offered to medicine, nursing, social work students, and chaplain learners. Although it had many positive outcomes, it mainly utilized online modules for didactic sessions. One study utilizing iCOPE assessed the learning of medical students during a clerkship with palliative care; however, this was focused solely on the medical student journey and not those of the other learners, who frequently spend time with the palliative care service.^5^

The Adult Palliative Care Program (APCP) at Michigan Medicine is a robust educational site that allows medical, nursing, pharmacy, social work, and chaplain learners of all levels of education to spend time with the clinical teams; learners participate in daily rounds, symptom management consults, goals of care conversations and complex end-of-life (EOL) patient care. The palliative care clinical teams are made up of physicians, nurse practitioners, social workers, a pharmacist, and a chaplain. Within this structure exists the Palliative Care Education Committee (PCEC). The PCEC is responsible for formalized educational efforts offered by the APCP and throughout the medical campus to extend the reach of palliative care education. There is a clear lack of formalized interprofessional education (IPE) within the clinical setting, both within the literature and in the APCP. The objective of this article is to describe the interprofessional team's needs assessment, efforts to address the identified needs, and preliminary results of a quality improvement initiative utilizing the plan-do-study-act (PDSA) model.^6,7^

## Design and Methods

A large descriptive study was previously conducted by members of the PCEC.^8,9^ This study determined that specific training for each discipline is needed, but common themes emerged across all disciplines, such as confidence in providing emotional support to patients and families, lack of confidence in providing continuity of care,^8^ and need for education on the topics of “cultural influences on care preferences, improving communication between patients/families and providers, education about the differences between palliative and EOL care, and increased competency of health providers in having EOL/goals-of-care discussions.”^9^

Given this information, the interprofessional team reviewed the APCP's current educational efforts across and within disciplines. It was noted that although the APCP offers unique educational experiences to learners, there was no formal training, learning objectives, dedicated time, or consistency in teaching method or educator. To address this, a group of interprofessional team members volunteered their time and applied for an educational grant offered through the University of Louisville, known as Interprofessional Education eXchange (IPEX). This grant offered an intensive on-site learning program followed by structured guidance from palliative care educators across several disciplines.

The design of the Michigan Medicine IPEX project was carefully constructed with the goal of providing a high-quality consistent learning experience, reaching the most learners, and placing minimal burden on the interprofessional team members involved. Currently, learners select palliative care as a shadowing, elective, fellowship or internship experience and are paired with a member of the learner's own discipline for the duration of their experience. Some learners shadow for only a four-hour time block and others elect to stay for one to four weeks at a time; some learners are accepted into a fellowship or internship and may work in palliative care for up to one full academic year. Learners come from all disciplines—medicine, pharmacy, nursing, social work, spiritual care, and health sciences—and may be at any place in their academic career—undergraduate, graduate, residency, and fellowship. This presented both a challenge and an opportunity—whereas short-term learners may only gain exposure to interprofessional collaboration, longer-term learners may have an opportunity to move through a more immersive experience and build competence in core palliative care skills.^10^ The goal was that all learners, regardless of clinical experience duration, would leave with some new knowledge or skills in interprofessional collaboration.

The interprofessional team reviewed literature pertaining to education in clinical palliative care settings and existing programs. The literature illustrated a lack of formalized efforts and dedicated time in the clinical setting.^1,5^ Formalized IPE programs are highly burdensome to clinicians who are already busy: reviewing reflective writings of each learner would be hugely time consuming,^5^ as would reviewing learner evaluations and making programmatic changes on a regular basis.^6^ iCOPE, a commonly used online learning module for palliative care, was not utilized for this project due to the focus on oncology; the team felt that it was too narrow of a focus, given the wide array of students who entered into the clinical learning experience.

**FIG. 1.**
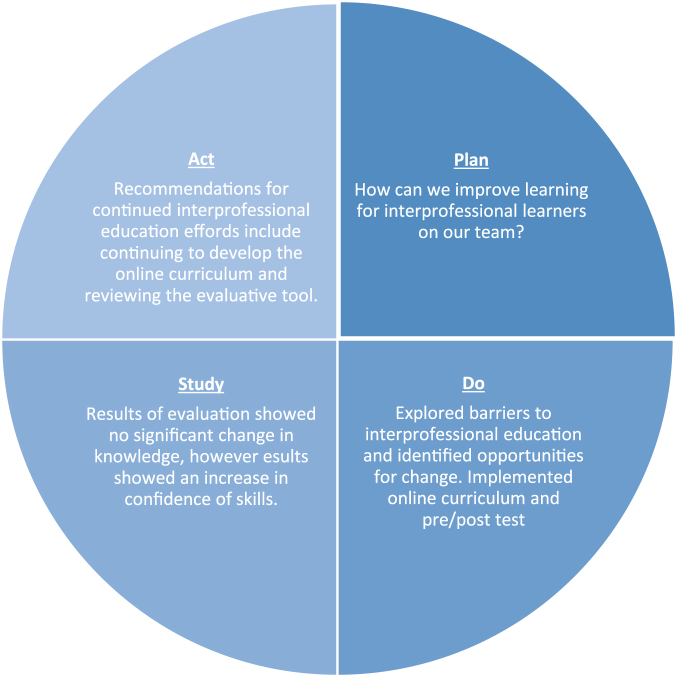
Plan, do, study, and act cycle.

**FIG. 2.**
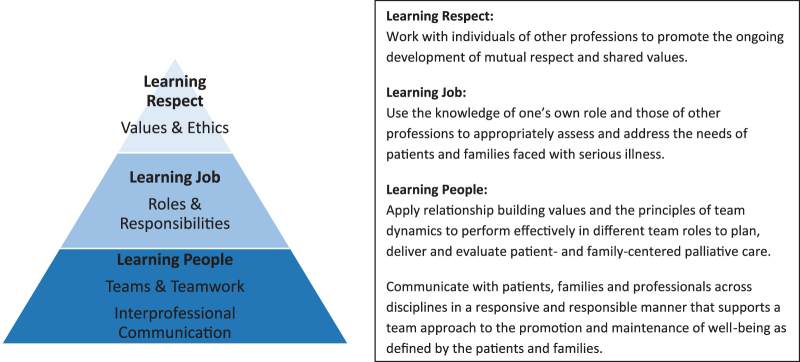
Thematic representation of learning curriculum and objectives.

The interprofessional team utilized the PDSA model^6,7^ to guide the project (Fig. 1). The goal of the clinical experience within palliative care was that all learners are able to apply the general principles of interprofessional palliative care to develop comprehensive care plans for patients and families faced with serious illnesses. By formalizing educational efforts, all learners should have been able to leave their clinical experience with a common foundation of knowledge in interprofessional palliative care that they may take forward and expand upon; this information was categorized into themes of learning “people,” learning “job,” and learning “respect” (Fig. 2). All learning outcomes and objectives were derived from the *Clinical Practice Guidelines for Quality Palliative Care*, published by the National Consensus Project and the National Coalition for Hospice and Palliative Care,^3^ and the *Core Competencies for Interprofessional Collaborative Practice*, published by the Interprofessional Education Collaborative.^11^ After examining these documents and supporting literature, a curriculum was built to outline the learning activities that would teach basic interprofessional palliative care knowledge, skills and attitudes, which would then be collectively evaluated by a knowledge test and a self-evaluation of perceived skills and attitudes. These learning outcomes should have been achieved by completing online learning modules and engaging in clinical experiences.

The online curriculum was developed for learners to utilize during their clinical rotation; the link to the online curriculum was included in the introductory e-mail. The curriculum was expected to take learners approximately three hours to complete; learners were not expected to complete this all at once, but instead to utilize this tool during times when they were not engaging in clinical work. The curriculum included both educational information, which was organized into modules in the form of written information and visual guides, and a case study. The aim of the curriculum was to facilitate asynchronous learning and ease the burden of teaching basic interprofessional and palliative care concepts for clinicians on the team; the curriculum also included a discussion board, which served as a way for asynchronous learners to share thoughts and ideas about their learning. The curriculum was initially utilized by selected interprofessional students, who provided feedback about the flow of the modules and the content offered; the curriculum was revised based on that feedback. The curriculum was then published for all learners.

A pretest was included in the standard introductory e-mail to learners one week before the start of their clinical experience. A post-test was e-mailed to learners after the last day of their clinical experience. In addition, printed copies were placed in envelopes and labeled as “Pre Test” and “Post Test,” along with a sign that welcomed learners and gave instructions to take the test if they had not already done so online; the envelopes and signs were placed in the area in the learner work room. All tests were anonymous; pre- and post-tests were not linked.

## Evaluation

The ideal tool to evaluate change in learners' knowledge, skills/behaviors, and attitudes of palliative care and interprofessional collaboration should measure in both a subjective and objective manner. Each tool discovered in the literature was evaluated (Table 1); however, no one tool was found to be adequate for interprofessional learners of clinical experiences.^12^ Therefore, the team opted to create a new evaluation tool (Appendix A1).

**Table 1.** Characteristics of Existing Interdisciplinary Education and Palliative Care Evaluation Tools

K/S/A = Knowledge, skills, and/or attitudes	Measures subjective K/S/A	Measures objective K/S/A	Evaluates discipline-specific K/S/A	Evaluates interprofessional K/S/A	Evaluates individual K/S/A	Evaluates team or group K/S/A	Evaluates palliative care K/S/A	Evaluates use of interprofessional K/S/A
Self-perceived EOL care competencies	X		X		X		X	
EOL professional caregiver survey	X		X		X		X	
PCQN	X		X		X		X	
PCKT	X		X		X		X	
Self-efficacy scale	X		X		X		X	
CTS	X		X		X		X	
ICAR	X		X		X		X	
JTOG	X		X		X		X	
iTOFT	X		X		X		X	

CTS, clinical teamwork scale; EOL, end-of-life; ICAR, interprofessional collaborator assessment rubric; iTOFT, individual teamwork observation and feedback tool; JTOG, Jefferson teamwork observation guide; PCKT, palliative care knowledge test; PCQN, palliative care quiz for nursing.

Using a pre- and post-test format, the newly developed evaluation tool measured the changes in learners' knowledge, skills/behaviors and attitudes, both objectively and subjectively, in regard to interprofessional palliative care. The pre- and post-tests differ only in introductory questions, as the post-test has additional questions, such as “Which palliative care experiences were you assigned to?” and “How long did you spend with the Adult Palliative Care Team?”

The test consisted of two parts—a knowledge section focused on palliative care interprofessional skills, and a skills/attitudes section focused on interprofessional competencies. The knowledge test consisted of 10 multiple-choice questions, which were derived from the Clinical Practice Guidelines for Quality Palliative Care (National Consensus Project, 2018). The skills and attitudes section consists of 17 Likert-scale questions, derived from the Core Competencies for Interprofessional Collaborative Practice (IPEC, 2016).

## Results

The pretest was completed by 43 learners, whereas the post-test was completed by 32 learners who rotated with the palliative care service for a period of six months. It is not known how many learners attended a clinical learning experience and did not complete surveys, as surveys were distributed through an anonymous link sent by an administrator. The majority of learners were medical (*n* = 25) and/or nursing (*n* = 25) on short clinical rotations (<3 weeks); three learners selected “other” as their discipline and responded with “genetic counseling,” “administration,” and “undergraduate.” The learners encompassed undergraduate (*n* = 19), graduate/medical students (*n* = 22), residents (*n* = 9), fellows (*n* = 2), and other (*n* = 1; undefined).

The learners encompassed undergraduate, graduate/medical students, residents, and fellows. The knowledge portion of the test showed no significant difference between proportion of learners answering correctly on the pre- versus the post-test for all questions. All questions except for three in the skills and attitudes portion of the test saw the proportion of learners who strongly agreed with the statements from pre- to post-test significantly increase (*p* < 0.05). For all questions, “strongly agree” was the desired outcome, as this selection indicated confidence in IPE skills. Three questions did not see improvement from pre- to post-test; these questions focused on self-reflection of team and self-performance, individuality as it relates to effective communication, and developing trusting relationships with patients. The latter two questions had high baseline agreement within the pretest.

## Discussion

*(Plan)* The inception of this project began with a desire to improve and standardize learning opportunities for interprofessional learners rotating with an inpatient adult palliative care service and reduce the burden of teaching for busy interprofessional clinicians. Therefore, an interprofessional team began to create an online curriculum for all learners to engage with and sought out an evaluation tool to measure the change in knowledge, skills, and attitudes among interprofessional learners; when they did not find a fitting tool, created one themselves.^12^ *(Do)* The pre-/post-test was administered primarily through e-mail and Qualtrics, with a few in paper form. Data were collected for a period of approximately six months. *(Study)* Disciplines represented by the survey mostly comprised medical and nursing learners. This does not encompass all of the learners that come through the APCP; it is unknown why learners social work was not represented in the study. It may be that these learners are on different rotations or set up their clinical experiences differently than the formalized process of medical and nursing learners.

The results of the knowledge pre- and post-test showed no significant difference, illustrating that either the knowledge of palliative care that learners could demonstrate was not changed or improved, or that the evaluation tool did not measure the knowledge that the learners were acquiring. The results also showed that learners are leaving more confident in most areas of interprofessional collaboration—an integral part of palliative care—than they were when they began their clinical experience. It is notable that no team members working with the APCP have scheduled time set aside for teaching; most of the teaching is done while working through cases and consults that must be addressed. Therefore, it is possible that learners are gaining skills to engage in the interprofessional work, rather than facts or traditional knowledge about palliative care.

The skills and attitudes questions that did not see improvement—self-reflection of team, self-performance—may be viewed as more challenging, expert-level skills, which may require more time than the learners were allotted. The skills and attitudes questions that had high baseline agreement within the pretest—effective communication and developing trusting relationships with patients—may illustrate some of the most basic skills students learn in their academic careers.

*(Act)* The results of this study indicate several areas for action. First, the online curriculum and knowledge test should be evaluated for content. Perhaps the information intended to be evaluated in the knowledge test is not highlighted in the online curriculum, or needs to be delivered in a different way. The online curriculum, at the time of this study, was in its first iteration and may need development, both in content and in online learning techniques (videos, *etc.*). In addition, it is not clear, based on this evaluation, if the learners are actually using the online curriculum—it may be beneficial in a future study to simply ask that question.

It is also possible that the evaluation tool did not adequately test the knowledge that was offered in the curriculum. The evaluation tool will also need to be reviewed for clarity; some questions may be misleading or unclear to learners new to the field.

Finally, although IPE may be present in many areas of health professions studies today, the skills required to effectively work on an interprofessional team are complex and advanced. It is not likely that the learners who are here for only a short amount of time are able to become experts in interprofessional collaboration, but there were encouraging results in interprofessional skills and attitudes. For that reason, it may be useful to examine the growth of skills and attitudes in learners who spend a longer amount of time working on an interprofessional team.

### Limitations

This study has several limitations. The inherent value and challenge of IPE is that all members are coming from different professional backgrounds and schools of thought; although the diversity of thought likely adds depth and wholeness to the experience, there are often differences in requirements for each health profession education program and that challenge was evident here. For example, medical students elect to spend two weeks in a clinical experience with palliative care, whereas master of social work students are only allowed 8–16 hours, and residents may be pulled unexpectedly to work in other areas based on hospital capacity. Nursing students may spend a number of days in a clinical experience, but they may not be consecutive, and pharmacy residents may be allowed a full uninterrupted month. This variation in clinical experience is very difficult to account for and profoundly limited the ways in which the curriculum could be used. The team's aim was to invite all interprofessional learners to engage in an in-person discussion, facilitated by a team member, to reflect and elaborate on the interprofessional experience; however, no common time could be identified. A discussion board was created to address this limitation of time, although it was not highly utilized.

The IPEX project was focused on intervention and development of a product that could be utilized within the clinical learning environment. For this reason, there were no pre-/post-tests done for learners before the online curriculum was established. Therefore, it is not clear what impact the online learning module had.

Finally, the tool used to evaluate change in knowledge, skills, and attitudes based on the clinical experience with palliative care has not been validated. Although tools were examined in depth by this interprofessional team,^12^ and this tool was developed out of that review, it has yet to be utilized in other clinical settings. Further exploration of this or other tools to evaluate interprofessional collaboration knowledge, skills, and attitudes in the clinical setting will be needed as this field continues to develop.

## Future Implications

Interprofessional collaboration will continue to grow as a means to effectively care for complex patients in an ever-changing health system; therefore, training in interprofessional skills for upcoming health professionals will be a necessary part of the education programs. The field requires continued exploration of educational initiatives that do not place excessive burden onto already busy clinicians and evaluation techniques that are tested and validated. One option may be using technology, which can provide modular and virtual learning experiences without requiring additional investment in faculty and staff time.

## Abbreviations Used

APCP: adult palliative care program
EOL: end of life
iCOPE: Interprofessional Curriculum for Oncology Palliative Care Education
IPE: interprofessional education
IPEX: interprofessional education exchange
PCEC: Palliative Care Education Committee
POSA: Plan-Do-Study Act

## Appendix A1. Pretest

**Pretest to Examine Knowledge, Skills, and Attitudes of Learners in an Interprofessional Palliative Care Experience**

**Introduction Questions**

Welcome to the Interprofessional Palliative Care Learning Experience! Before you begin, we would like to understand your baseline knowledge about interprofessional collaboration and palliative care. Please complete the following survey before your first day with the palliative care team. This survey is expected to take <10 minutes to complete.

**Q1 Describe the role of palliative care, as you would to another health care team member**:
_______________________________________ _______________________________________ _______________________________________
**Q2 What prior experience do you have with palliative care?**
 *Check all that apply*
□ Personal
□ Professional
□ No experience
**Q3 What is your discipline?**
○ Physician/medicine
○ Pharmacy
○ Nursing
○ Chaplain
○ Social work
○ Other _______________________
**Q4 What is your level of experience?**
○ Undergraduate student
○ Graduate student/medical student
○ Resident
○ Fellow
○ Other ______________________________
**Q5 What is your age?**
○ 18–24
○ 25–29
○ 30–34
○ 35–39
○ 40–44
○ 45–49
○ 50–54
○ 55–60
○ 60+

**Knowledge Assessment**

The following questions are meant to assess your knowledge of core palliative care and interprofessional collaboration principles. Please select the single best answer for each question, unless otherwise directed.

**Q1 Where can patients receive palliative care?** *Check all that apply.*
□ Nursing/assisted living facilities
□ Private residences/homes
□ Acute care facilities (hospitals)
□ Hospice facilities
□ Outpatient clinics
□ Long-term acute care facilities
**Q2 High-functioning interprofessional teams encourage what of all team members?**
○ Interprofessional-specific education
○ Research initiatives
○ Discipline-specific credentialing and certification
○ National interprofessional education (IPE) training
**Q3 Which of the following are examples of active listening?**
○ Avoiding nonverbal communication
○ Limiting eye contact
○ Offering reflective statements
○ Asking open-ended questions
**Q4 All members of the palliative care team should receive training in:** *Check all that apply.*
□ Risk assessment for opioid use/abuse
□ Use of opioids for pain management
□ Monitoring for signs of opioid abuse and diversion
□ Use of alternative therapies for pain management
**Q5 A thorough palliative care assessment should include:** *Check all that apply.*
□ A determination of decision-making capacity, or identification of the person with legal decision-making authority
□ Social determinate of health, including vulnerabilities of finances, housing, nutrition, and safety
□ A discussion of home layout, including number of stairs
□ Patient and family understanding of the serious illness, goals of care, and treatment preferences
**Q6 Pain can be addressed with which of the following modalities?**
*Check all that apply.*
□ Medication
□ Surgical intervention
□ Behavioral intervention
□ Psychotherapy
□ Acupuncture and massage
**Q7 Check the most appropriate answer: ____ can influence the experience of illness, reporting of symptoms, preferences for treatment and the decision-making process.**
○ Age
○ Culture
○ Gender
○ Marital status
**Q8 Effective symptom management requires attention to the physical, emotional, spiritual, and cultural factors, as well as ____, which contributes to total pain and suffering associated with serious illness.**
○ Gender
○ Treatment preferences
○ Social determinates of health
○ Insurance and financial status
**Q9 The palliative care team's goal for all patients is to maximize:** *Check all that apply.*
□ Patient safety
□ Family autonomy
□ Sense of control
□ Life expectancy
**Q10 Social determinants of health, which have a strong and sometimes overriding influence on patients with serious illness, may include:** *Check all that apply.*
□ Financial vulnerability
□ Housing
□ Nutrition
□ Safety
□ Social class

**Skills and Attitudes Evaluation**

The following questions are meant to assess your skills and attitudes related to interprofessional collaboration and palliative care. Please select one answer to rate your agreement for each statement.

**Q1 I am able to describe the process of group dynamics, team development, and the roles and practices of effective teams.**
○ Strongly agree
○ Somewhat agree
○ Neither agree nor disagree
○ Somewhat disagree
○ Strongly disagree
**Q2 I can successfully engage health and other professionals in shared patient- and family-centered problem solving, and share accountability for patient outcomes.**
○ Strongly agree
○ Somewhat agree
○ Neither agree nor disagree
○ Somewhat disagree
○ Strongly disagree
**Q3 I am able to integrate the knowledge and experience of health and other interprofessional team members to inform health and care decisions, while respecting patient and family values and priorities/preferences for care.**
○ Strongly agree
○ Somewhat agree
○ Neither agree nor disagree
○ Somewhat disagree
○ Strongly disagree
**Q4 I can manage disagreements about values, roles, goals, and actions that arise between interprofessional team members, patients, and families.**
○ Strongly agree
○ Somewhat agree
○ Neither agree nor disagree
○ Somewhat disagree
○ Strongly disagree
**Q5 I am able to reflect on individual and team performance and improvement. I know what I need to improve upon and *how* I can work on improvement.**
○ Strongly agree
○ Somewhat agree
○ Neither agree nor disagree
○ Somewhat disagree
○ Strongly disagree
**Q6 I am able to actively listen for understanding, and integrate ideas and opinions of interprofessional team members.**
○ Strongly agree
○ Somewhat agree
○ Neither agree nor disagree
○ Somewhat disagree
○ Strongly disagree
**Q7 I can successfully communicate with patients, families, and health care team members in a form that is understandable to those receiving it.**
○ Strongly agree
○ Somewhat agree
○ Neither agree nor disagree
○ Somewhat disagree
○ Strongly disagree
**Q8 I am comfortable expressing my knowledge and opinions to interprofessional team members involved in patient care with confidence, clarity, and respect, working to ensure a common understanding.**
○ Strongly agree
○ Somewhat agree
○ Neither agree nor disagree
○ Somewhat disagree
○ Strongly disagree
**Q9 I am comfortable eliciting timely, sensitive, and instructive feedback about my own performance on the interprofessional team and respond respectfully as a team member provides feedback.**
○ Strongly agree
○ Somewhat agree
○ Neither agree nor disagree
○ Somewhat disagree
○ Strongly disagree
**Q10 I appreciate and understand how each person's own uniqueness (experience level, expertise, culture, power, and hierarchy within the health team) contributes to effective communication, conflict resolution, and relationships.**
○ Strongly agree
○ Somewhat agree
○ Neither agree nor disagree
○ Somewhat disagree
○ Strongly disagree
**Q11 I am able to effectively describe the roles and responsibilities of the interprofessional team clearly to patients, families, and other professionals.**
○ Strongly agree
○ Somewhat agree
○ Neither agree nor disagree
○ Somewhat disagree
○ Strongly disagree
**Q12 I can recognize my own limitations in scope of practice, skills, knowledge, and abilities. I know when to call in another member of my interprofessional team to help me.**
○ Strongly agree
○ Somewhat agree
○ Neither agree nor disagree
○ Somewhat disagree
○ Strongly disagree
**Q13 I am able to collaborate with team members to clarify each members' responsibility in executing unique components of a treatment plan that is safe, timely efficient, effective, and equitable.**
○ Strongly agree
○ Somewhat agree
○ Neither agree nor disagree
○ Somewhat disagree
○ Strongly disagree
**Q14 I am able to incorporate patient's and family's perceptions of illness, suffering, and well-being, and engage with the interprofessional team, to address patient and family needs.** Page 7 of 7
○ Strongly agree
○ Somewhat agree
○ Neither agree nor disagree
○ Somewhat disagree
○ Strongly disagree
**Q15 I am able to complete an interprofessional assessment of total suffering for and with patients and families with serious illness.**
○ Strongly agree
○ Somewhat agree
○ Neither agree nor disagree
○ Somewhat disagree
○ Strongly disagree
**Q16 I am able to develop a trusting relationship with patients, families, and other team members with an emphasis on honesty and integrity.**
○ Strongly agree
○ Somewhat agree
○ Neither agree nor disagree
○ Somewhat disagree
○ Strongly disagree
**Q17 I am comfortable managing ethical dilemmas that arise in the practice of interprofessional palliative care.**
○ Strongly agree
○ Somewhat agree
○ Neither agree nor disagree
○ Somewhat disagree
○ Strongly disagree
